# A breath of fresh air: Validity and reliability of a Portuguese version of the Multidimensional Dyspnea Profile for patients with COPD

**DOI:** 10.1371/journal.pone.0215544

**Published:** 2019-04-30

**Authors:** Letícia F. Belo, Antenor Rodrigues, Ana Paula Vicentin, Thaís Paes, Larissa Araújo de Castro, Nidia A. Hernandes, Fabio Pitta

**Affiliations:** 1 Laboratory of Research in Respiratory Physiotherapy (LFIP), Department of Physiotherapy, Universidade Estadual de Londrina (UEL), Londrina, Paraná, Brazil; 2 Department of Rehabilitation Sciences, Katholieke Universiteit Leuven, Leuven, Belgium; Universiteit van Amsterdam, NETHERLANDS

## Abstract

**Aim:**

To provide a Portuguese version of the Multidimensional Dyspnea Profile (MDP), investigating its validity and reliability in Brazilian patients with COPD.

**Methods:**

This was a cross-sectional study for translation and linguist validation of the Portuguese MDP version for patients with COPD. The process occurred according to the protocol of Mapi Research Trust, Lyon, France. Three scores of MDP were used for the analysis: the immediate unpleasantness of dyspnea (A1); the “immediate perception domain” (S) (sum of A1 plus the sensory descriptors) and the “emotional response domain” (A2) (sum of the emotional descriptors). The questionnaires COPD assessment Test (CAT), Hospital Anxiety and Depression scale (HADS) and Medical Research Council scale (MRC) were used as anchors to investigate MDP’s validity. Internal consistency was assessed with Cronbach’s alpha. Test–retest reliability was assessed with intraclass correlation coefficient (ICC) and concurrent validity was assessed with Spearman correlation coefficients.

**Results:**

Thirty patients with moderate-severe COPD were studied for MDP’s validation analysis (43% male, 63±8years, body mass index [BMI] 27±6Kg/m^2^, forced expiratory volume in the first second [FEV_1_] 48±15%predicted, six-minute walking test [6MWT] 464±84m and 84±16%predicted), whereas 10 patients were excluded from the test-retest reliability analysis due to missing data, resulting in a sample of 20 subjects for this purpose (50% male, 62±8years, BMI 27±6Kg/m^2^, FEV_1_ 48±15%predicted, 6MWT 452±93m and 82±19%predicted). Both samples were similar regarding general characteristics (*P*>0,05 for all variables). MDP presented strong correlations, i.e., ICC intra-rater: A1: 0.77 (0.48–0.90), S: 0.78 (0.52–0.91), and A2: 0.85 (0.66–0.94), with high internal consistency (Cronbach's α 0.86, 0.88 and 0.92 respectively); and ICC inter-rater: A1: 0.74 (0.46–0.89), S: 0.75 (0.48–0.89) and A2: 0.91 (0.78–0.96) with Cronbach's α 0.85, 0.86 and 0.95 respectively.

**Conclusion:**

The Portuguese version of the MDP is the first valid and reliable instrument to assess dyspnea multidimensionally in Portuguese-speaking patients with COPD.

## Introduction

Dyspnea is a major disabling symptom reported by patients with chronic obstructive pulmonary disease (COPD), described as a subjective experience, derived from interactions among multiple physiological, psychosocial, and environmental factors [1]. Noteworthy, the majority of available instruments in Portuguese assesses dyspnea only in a one-dimensional approach [2,3]. Therefore, an instrument in Portuguese language which is able to assess and distinguish multiple aspects of dyspnea is necessary.

The multidimensional dyspnea profile (MDP) is an instrument indicated for this purpose [4–7]. It assesses immediate respiratory discomfort, qualities of the breathlessness, and emotional responses [4,6]. It is already known that MDP is sensitive to detect changes in dyspnea sensation evoked by different physiologic stimulus [6]. Furthermore, the English and French versions of MDP were already shown to be valid and reproducible for clinical and laboratory assessments, also presenting excellent internal reliability (Cronbach's α ≥ 0.84) [4,5,7]. However, a Portuguese version of this instrument is not yet available, hindering its use in Portuguese-speaking countries. Therefore, the aim of this study was to provide a Portuguese version of MDP, investigating its validity and reliability in Brazilian patients with COPD.

## Material and methods

This was a cross-sectional study involving the translation and linguist validation of the MDP. The Portuguese version has been developed by three of the present researchers (LFB, LAC and NAH) in collaboration with Robert Banzett and Mapi Research Trust, Lyon, France (for information about permission to use the MDP, please access *https*:*//eprovide*.*mapi-trust*.*org*), according to standard process of forward and backward translations, followed by cognitive interviews with five patients with previous diagnosis of chronic respiratory diseases (COPD, bronchiectasis and asthma) (S1 and S2 Appendix). The process was undertaken similarly to the French version [7], and it was not necessary to adapt or withdraw any item of the instrument.

Patients were recruited during the baseline evaluation before taking part in a pulmonary rehabilitation program, and only pre-rehabilitation data were used for the present study. COPD diagnosis was done by spirometry, according to international criteria as described in the Global Initiative for Obstructive Lung Disease (GOLD) [8]. In addition to the diagnosis of COPD, other inclusion criteria were: one-month clinical stability and absence of severe and/or unstable cardiac disease and musculoskeletal comorbidities that could interfere in the assessments. Exclusion criteria were the occurrence of osteoneuromuscular complications or acute exacerbation during the assessment period. Patients who did not perform the second or third MDP assessment were excluded from the reliability analysis. The study was approved by the Ethics Committee on Research Involving Human Beings of State University of Londrina (number: 1.887.424) and all patients provided informed consent.

The MDP was applied in three different time-points: day one and day two 24 hours apart, by different raters, and day three, by the first rater, one to two weeks after the first evaluation. The focus period was established as breathlessness during activities of daily living (ADL) on the past 2 weeks. The MDP consists of 11 items evaluating sensory and affective dimensions of dyspnea. One item assesses the immediate unpleasantness of dyspnea (A1) on a 0–10 visual numerical scale anchored by “neutral” (0) and “unbearable” (10). Five items assess dyspnea’s sensory dimension and five items assess affective dimension of dyspnea, in terms of quality and intensity (on a scale of 0–10). Further, two scores are calculated: an “immediate perception domain” score (S), corresponding to the sum of A1 intensity plus intensities of the five sensory descriptors; and an “emotional response domain” score (A2), corresponding the sum of the five emotional descriptors [4,5].

Besides the assessment of dyspnea by the MDP, patients were assessed regarding pulmonary function (spirometry) and exercise capacity (6-minute walk test [6MWT]) following international guidelines and local reference values [9–12]. Furthermore, health status (COPD assessment Test—CAT)[3], anxiety and depression (Hospital Anxiety and Depression scale–HADS)[13] and dyspnea in daily life (Medical Research Council scale–MRC)[2] were also assessed and used as anchors.

Statistical analyses were performed with the SPSS Statistical Package 21.0 (IBM SPSS Statistics, Chicago, IL, USA). Normality in data distribution was evaluated using the Shapiro-Wilk test. Data were described as mean±standard deviation or median [interquartile range 25%-75%]. The concurrent validity was assessed by correlations of the MDP domains with CAT, HADS and MRC scores, using the Spearman correlation coefficient. Internal consistency was assessed by Cronbach’s α. Inter- and intra-rater test-retest reliability was evaluated between all items and dimensions of the MDP using intraclass coefficient correlation (ICC). For Cronbach’s α and ICC, values greater than 0.70 were considered satisfactory [14]. Significance level was set at *P*<0.05.

Sample size was calculated from the values proposed by Hulley et al.[15] based on the study by Silva et al.[3], expecting a minimum correlation of 0.60 between MDP and CAT, considering a two-sided alpha value of 0.05 and 80% of power. Hence, a minimum of 19 individuals were required.

## Results

Thirty patients with COPD were included in the validation analysis (43% male, 63±8years, body mass index [BMI] 27±6Kg/m^2^, forced expiratory volume in the first second [FEV_1_] 48±15%predicted, 6MWT 464±84m and 84±16%predicted). Ten patients were excluded from the reliability analysis due to the unavailability of data from the second and/or third MDP application upon these patients’ request to be dismissed from this assessment. This resulted in a sample of 20 subjects for this purpose (50% male, 62±8years, BMI 27±6Kg/m^2^, FEV_1_ 48±15%predicted, 6MWT 452±93m and 82±19%predicted). Both samples were similar regarding general characteristics (*P*>0.05 for all). No patient reported exacerbation of symptoms and/or hospitalization during the focal period (data evaluated by a general questionnaire of anthropometric and exacerbation data).

**Fig 1:**
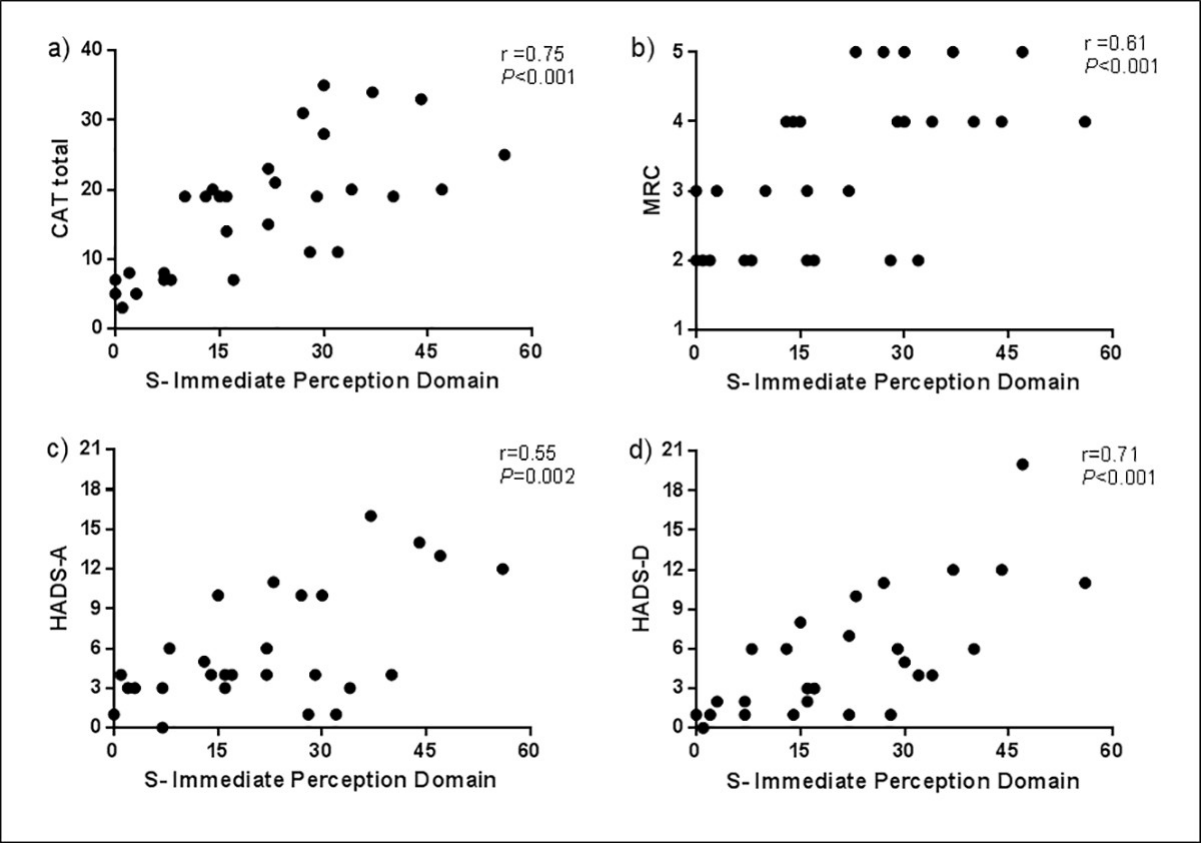
Correlations between the immediate perception domain (S) and the established anchors. (a) CAT: COPD Assessment Test; (b) MRC: Medical Research Council; (c) HADS-A: Hospital Anxiety and depression scale–Anxiety subscale; (d)HADS-D: Hospital Anxiety and depression scale–Depression subscale.

**Fig 2:**
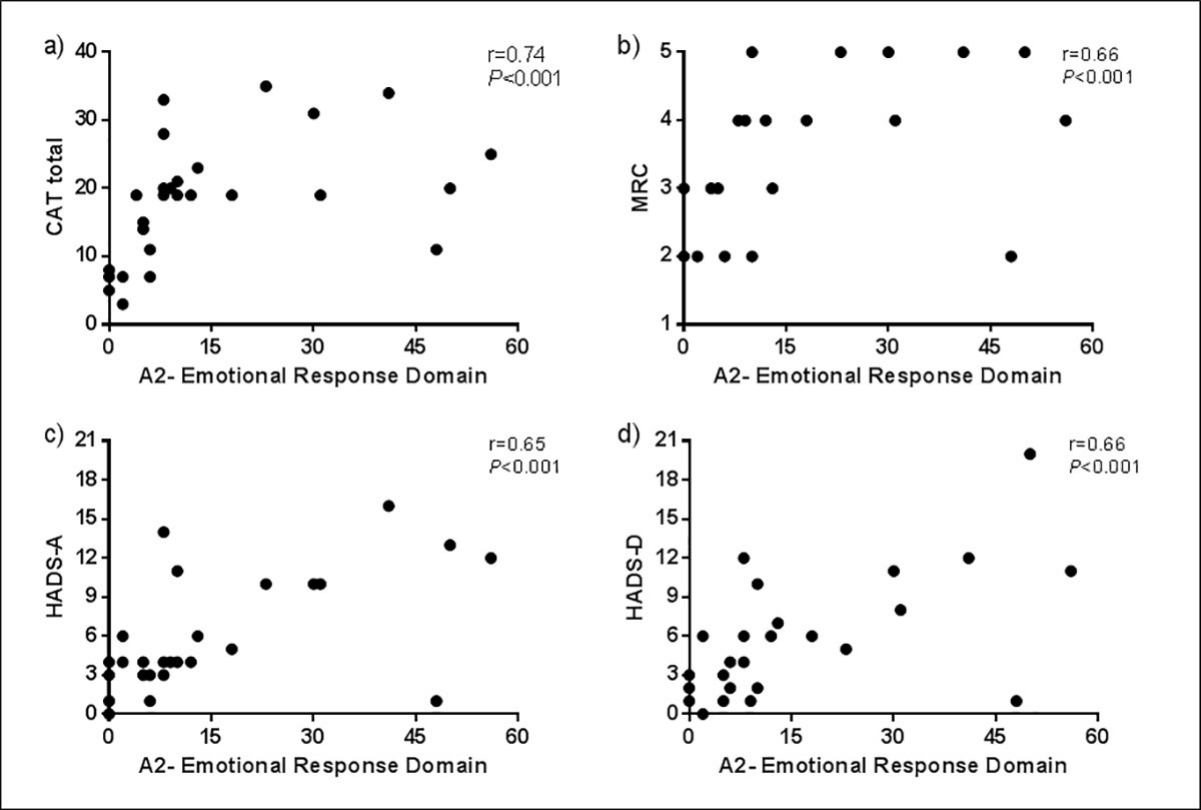
Correlations between the emotional response domain (A2) and the established anchors. (a) CAT: COPD Assessment Test; (b) MRC: Medical Research Council; (c) HADS-A: Hospital Anxiety and depression scale–Anxiety subscale; (d) HADS-D: Hospital Anxiety and depression scale–Depression subscale.

According to the concurrent validity, the Portuguese version of the MDP was moderately to strongly correlate with the questionnaires CAT, HADS and MRC, as shown in Table 1, Fig 1 and Fig 2.

**Table 1:** Concurrent validity of the Multidimensional Dyspnea Profile (MDP) with clinical instruments.

MDP Variables	CAT Total	HADS Anxiety	HADS Depression	MRC
A1- Immediate umpleseanteness	0.62*	0.68*	0.71*	0.55*
Breathing effort	0.59*	0.33	0.43*	0.44*
Air hunger	0.61*	0.45*	0.53*	0.53*
Chest tightness	0.55*	0.48*	0.59*	0.48*
Mental effort	0.63*	0.64*	0.64*	0.63*
Breathing a lot	0.37*	0.04	0.25	0.13
**S- Immediate Perception Domain**	0.75*	0.55*	0.71*	0.61*
Depression	0.44*	0.54*	0.52*	0.53*
Anxiety	0.75*	0.58*	0.66*	0.62*
Frustration	0.54*	0.52*	0.42*	0.52*
Anger	0.42*	0.44*	0.40*	0.34
Fear	0.26	0.32	0.52*	0.35
**A2- Emotional Domain**	0.74*	0.65*	0.66*	0.66*

Spearman correlation coefficient

*P<0.05

CAT: COPD assessment Test; HADS: Hospital Anxiety and Depression scale; MRC: Medical Research Council scale

All dimensions of the MDP demonstrated to be reliable independently of different raters and had good internal consistency. Intra-rater ICCS were: A1: 0.77 (0.48–0.90), S: 0.78 (0.52–0.91), and A2: 0.85 (0.66–0.94), and Cronbach's α of 0.86, 0.88 and 0.92, respectively. Inter-rater ICCs were: A1: 0.74 (0.46–0.89), S: 0.75 (0.48–0.89) and A2: 0.91 (0.78–0.96), with Cronbach's α of 0.85, 0.86 and 0.95, respectively.

## Discussion

Similarly to the French version [7], the moderate-to-strong correlations between the MDP Portuguese version and *a priori* established anchors (i.e., CAT, HADS and MRC) (Table 1) demonstrate that this version was valid to assess dyspnea in patients with COPD, strongly endorsing its use. Notably, concurrent validity was carried out against these three instruments since they are widely used in COPD and measure dimensions similar to the MDP’s composition.

Interestingly, the description “breathing a lot” was poorly correlated with all variables (Table 1). This may have happened because only 19% of patients reported this descriptor, hindering its correlation with other variables. Likewise, Banzett et al. [6] unveil this sensation as being reported during induced-hyperpnea in heathy subjects. Indeed, the focus period in the present study was based on ADL (i.e., lower ventilatory burden); this could have mitigated this description by our patients. Moreover, CO_2_ desensitization can also be hypothesized as a possible explanation [1,4].

Test-retest reliability analysis showed that MDP is highly reliable, independently whether performed by the same or a different rater. This reinforces the fact that it is not necessary the same rater in different evaluations, but another trained rater who knows the instrument may also re-apply it, simplifying the MDP application.

These results have to be taken into account in light of study’s strengths and limitations. Firstly, the samples were not the same for two analyses (validity and reliability), however both were larger than the minimum required sample size (i.e., 19 subjects), and these samples presented no differences between them concerning general characteristics. Second, the focus period of the present study, which was ADL in the last two weeks as well as the intervals between time-points 1–2 and 3 (one to two weeks), can be seen as time periods with risk of change in symptoms; however, this study followed the recommendations of specific guidelines for reliability studies [16]. Moreover, patients’ breathlessness was not affected in the study course since none of the patients experienced COPD exacerbations or other acute conditions during the study’s period.

Not less important, despite translation was done according to the Portuguese language spoken in Brazil; we believe there is no limitation for its use in any Portuguese-speaking countries. Whether necessary, minor changes are possible without changing sentences’ meaning. Furthermore, the MDP was not developed for a specific disease. Thus, as for other languages [5–7], the Portuguese version can be used without adaptations for a wide spectrum of disease conditions, although specific validation is required. In addition, a minimal important difference for the MDP has not been established, and this is an open field for future research.

Therefore, according to the present results it is possible to affirm that the Portuguese version of the MDP is the first valid and reliable instrument to assess dyspnea multidimensionally in Portuguese-speaking patients with COPD.

## Supporting information

S1 Appendix Multidimensional Dyspnea Profile: Portuguese and English versions. (PDF)

S2 Appendix Translation process and linguistic validation of Portuguese version of the Multidimensional Dyspnea Profile. (PDF)
