# Supplementary material for: A breath of fresh air: Validity and reliability of a Portuguese version of the Multidimensional Dyspnea Profile for patients with COPD
Source: PLoS One. 2019 Apr 30;14(4):e0215544. doi: 10.1371/journal.pone.0215544 (PMC6490879; doi:10.1371/journal.pone.0215544)
Supplement: S1 Appendix — (PDF) [file pone.0215544.s001.pdf]

Supporting information to:

A breath of fresh air: validity and reliability of a Portuguese version of the Multidimensional Dyspnea Profile for patients with COPD.

Authors: Letícia F. Belo[1], Antenor Rodrigues[1,2], Ana Paula Vicentin[1], Thaís Paes[1], Larissa A. de Castro[1], Nidia A. Hernandes[1], Fabio Pitta[1*].

[1]Laboratory of Research in Respiratory Physiotherapy (LFIP), Department of Physiotherapy, Universidade Estadual de Londrina (UEL), Londrina, Paraná, Brazil.

[2]Department of Rehabilitation Sciences, Katholieke Universiteit Leuven, Leuven, Belgium.

S1 Appendix. Multidimensional Dyspnea Profile: Portuguese and English versions.

Perfil Multidimensional de Dispneia pag 1 de 9 nome/identificação_____________________data e hora

PERFIL MULTIDIMENSIONAL DE DISPNEIA

©2011 R.B.Banzett. Todos os Direitos Reservados.

Roteiro para a primeira aplicação:

O objetivo deste questionário é nos ajudar a entender como você sente a sua respiração.

Não existem respostas certas ou erradas. Nós gostaríamos de saber o que você tem para nos dizer sobre a sua respiração.

Nesta página, nós lhe pedimos que nos diga o quão desagradável você sente a sua respiração. Na próxima página, nós lhe perguntaremos sobre a intensidade ou força das suas sensações ao respirar. A distinção entre esses dois aspectos relacionados à sensação ao respirar pode ser mais facilmente entendida se você se imaginar ouvindo um som, como de um rádio. Conforme o volume do som aumenta, eu posso lhe perguntar o quão alto isso lhe parece ou o quão desagradável é ouvir esse som. Por exemplo, uma música que você não gosta pode ser desagradável mesmo quando o volume está baixo, e pode se tornar ainda mais desagradável se o volume do som for aumentado. Da mesma forma, uma música que você gosta não se tornará desagradável mesmo se o volume do som for aumentado.

<u>Escala A1</u>

Use essa escala para graduar o **desagrado ou desconforto** da sua sensação ao respirar, quão **ruim** é (era) sua sensação ao respirar.

Por favor, foque durante o seguinte período___________________

←	←	0	1	2	3	4	5	6	7	8	9	10
AGRADÁVEL		NEUTRO										INSUPORTÁVEL

 nome/identificação_____________________data e hora

Característica da Sensação (CS): Escolha

Abaixo encontram-se frases ou termos organizados em grupos com significado similar.

Passo 1: Marque cada grupo que descreve como é (era) a sua sensação ao respirar durante _____________________(indicar o período de tempo).

Passo 2: Por favor, marque também *um* grupo que melhor descreve como é (era) sua sensação ao respirar.

	**Passo 1**		**Passo 2**
Se *QUALQUER* termo no grupo se aplicar, escolha esse grupo.	NÃO SE APLICA	APLICA-SE	GRUPO QUE MELHOR DESCREVE
Minha respiração requer trabalho muscular ***ou*** esforço			
Eu não respiro ar suficiente ***ou*** eu me sinto sufocado ***ou*** eu sinto fome de ar			
Sinto meu peito e pulmões apertados ***ou*** restritos			
Minha respiração requer esforço mental ***ou*** concentração			
Eu estou respirando demais			

Característica da Sensação (CS): Escalas

Use essas escalas para quantificar como é (era) a intensidade das suas sensações ao respirar (como a altura do som, independente do fato da sensação ser agradável ou desagradável. Por exemplo, uma sensação pode ser intensa sem ser desagradável).

Por favor, foque durante o seguinte período_____________________

Se QUALQUER termo no grupo se aplicar, classifique esse grupo.	**NENHUMA**										**O MAIS INTENSO QUE EU POSSO IMAGINAR**
Minha respiração requer trabalho muscular ***ou*** esforço	0	1	2	3	4	5	6	7	8	9	10
Eu não respiro ar suficiente ***ou*** eu me sinto sufocado ***ou*** eu sinto fome de ar	0	1	2	3	4	5	6	7	8	9	10
Sinto meu peito e pulmões apertados ***ou*** restritos	0	1	2	3	4	5	6	7	8	9	10
Minha respiração requer esforço mental ***ou*** concentração	0	1	2	3	4	5	6	7	8	9	10
Eu estou respirando demais	0	1	2	3	4	5	6	7	8	9	10
Outro*	0	1	2	3	4	5	6	7	8	9	10

*Caso ache necessário, você pode adicionar descrições da sua sensação ao respirar.

 nome/identificação______________________data e hora

Escalas A2

Quando você sente que a sua respiração não está normal, você pode experimentar emoções ou “sentimentos”. Usando as escalas abaixo, por favor, conte-nos sobre como suas sensações ao respirar fizeram você se sentir – classifique zero para qualquer emoção que você não tenha sentido.

Por favor, foque nas sensações durante o seguinte período______________________.

	NENHUMA										O MAIS INTENSO QUE EU POSSO IMAGINAR
Deprimido	0	1	2	3	4	5	6	7	8	9	10
Ansioso	0	1	2	3	4	5	6	7	8	9	10
Frustrado	0	1	2	3	4	5	6	7	8	9	10
Com raiva	0	1	2	3	4	5	6	7	8	9	10
Com medo	0	1	2	3	4	5	6	7	8	9	10
Outro?	0	1	2	3	4	5	6	7	8	9	10

MULTIDIMENSIONAL DYSPNEA PROFILE

©2011 R.B.Banzett. All Rights Reserved.

Script for first time use:

The purpose of this questionnaire is to help us understand how your breathing feels.

There are no right or wrong answers. We want to know what you tell us about your own breathing.

On this page we ask you to tell us how unpleasant your breathing feels. On a later page we will ask you about the intensity or strength of your breathing sensations. The distinction between these two aspects of breathing sensation might be made clearer if you think of listening to a sound, such as a radio. As the volume of the sound increases, I can ask you how loud it sounds or how unpleasant it is to hear it. For example, music that you hate can be unpleasant even when the volume is low, and will become more unpleasant as the volume increases; music that you like will not be unpleasant, even when the volume increases.

A1 Scale

Use this scale to rate the **unpleasantness or discomfort** of your breathing sensations, how **bad** your breathing feels [felt].

Please focus on the period when____________________

← ←	0	1	2	3	4	5	6	7	8	9	10
PLEASANT	NEUTRAL										UNBEARABLE

SQ choice

Below are phrases or terms arranged in groups of similar meaning.

Step 1: Check each group that describes how your breathing feels [felt] during ______________________(indicate focus period).

Step 2: Please also mark *one* group that most accurately describes how your breathing feels [felt].

	**Step 1**		**Step 2**
If *ANY* term in the group applies, choose that group.	DOES NOT APPLY	DOES APPLY	MOST ACCURATELY DESCRIBES
My breathing requires muscle work ***or*** effort			
I am not getting enough air ***or*** I am smothering ***or*** I feel hunger for air			
My chest and lungs feel tight ***or*** constricted			
My breathing requires mental effort ***or*** concentration			
I am breathing a lot			

SQ Scales

Use these scales to rate the intensity of the breathing sensations you feel [felt] (like the loudness of sound, regardless of whether the sensation is pleasant or unpleasant; for example a sensation could be intense without being unpleasant.)

Please focus on the period when ______________________

If ANY term in the group applies, rate that group.	**NONE**										AS INTENSE AS I CAN IMAGINE
My breathing requires muscle work ***or*** effort	0	1	2	3	4	5	6	7	8	9	10
I am not getting enough air ***or*** I am smothering ***or*** I feel hunger for air	0	1	2	3	4	5	6	7	8	9	10
My chest and lungs feel tight ***or*** constricted	0	1	2	3	4	5	6	7	8	9	10
My breathing requires mental effort ***or*** concentration	0	1	2	3	4	5	6	7	8	9	10
I am breathing a lot	0	1	2	3	4	5	6	7	8	9	10
Other*	0	1	2	3	4	5	6	7	8	9	10

*If you need to, you can add additional descriptions of your breathing sensations.

A2 Scales

When your breathing doesn't feel normal, you may experience emotions or 'feelings'. Using the scales below, please tell us about how your breathing sensations made you feel – rate zero for any emotion you did not feel.

Please focus on feelings during the period when ________________________.

	NONE										THE MOST I CAN IMAGINE
Depressed	0	1	2	3	4	5	6	7	8	9	10
Anxious	0	1	2	3	4	5	6	7	8	9	10
Frustrated	0	1	2	3	4	5	6	7	8	9	10
Angry	0	1	2	3	4	5	6	7	8	9	10
Afraid	0	1	2	3	4	5	6	7	8	9	10
Other?	0	1	2	3	4	5	6	7	8	9	10
