# Supplementary material for: A breath of fresh air: Validity and reliability of a Portuguese version of the Multidimensional Dyspnea Profile for patients with COPD
Source: PLoS One. 2019 Apr 30;14(4):e0215544. doi: 10.1371/journal.pone.0215544 (PMC6490879; doi:10.1371/journal.pone.0215544)
Supplement: S2 Appendix — (PDF) [file pone.0215544.s002.pdf]

**Supporting information to:**

# A breath of fresh air: validity and reliability of a Portuguese version of the Multidimensional Dyspnea Profile for patients with COPD.

**Authors:** Letícia F. Belo[1], Antenor Rodrigues[1,2], Ana Paula Vicentin[1], Thaís Paes[1], Larissa A. de Castro[1], Nidia A. Hernandes[1], Fabio Pitta[1*].

[1]Laboratory of Research in Respiratory Physiotherapy (LFIP), Department of Physiotherapy, Universidade Estadual de Londrina (UEL), Londrina, Paraná, Brazil.

[2]Department of Rehabilitation Sciences, Katholieke Universiteit Leuven, Leuven, Belgium.

**S2 Appendix. Translation process and Linguistic validation of Portuguese version of the Multidimensional Dyspnea Profile.**

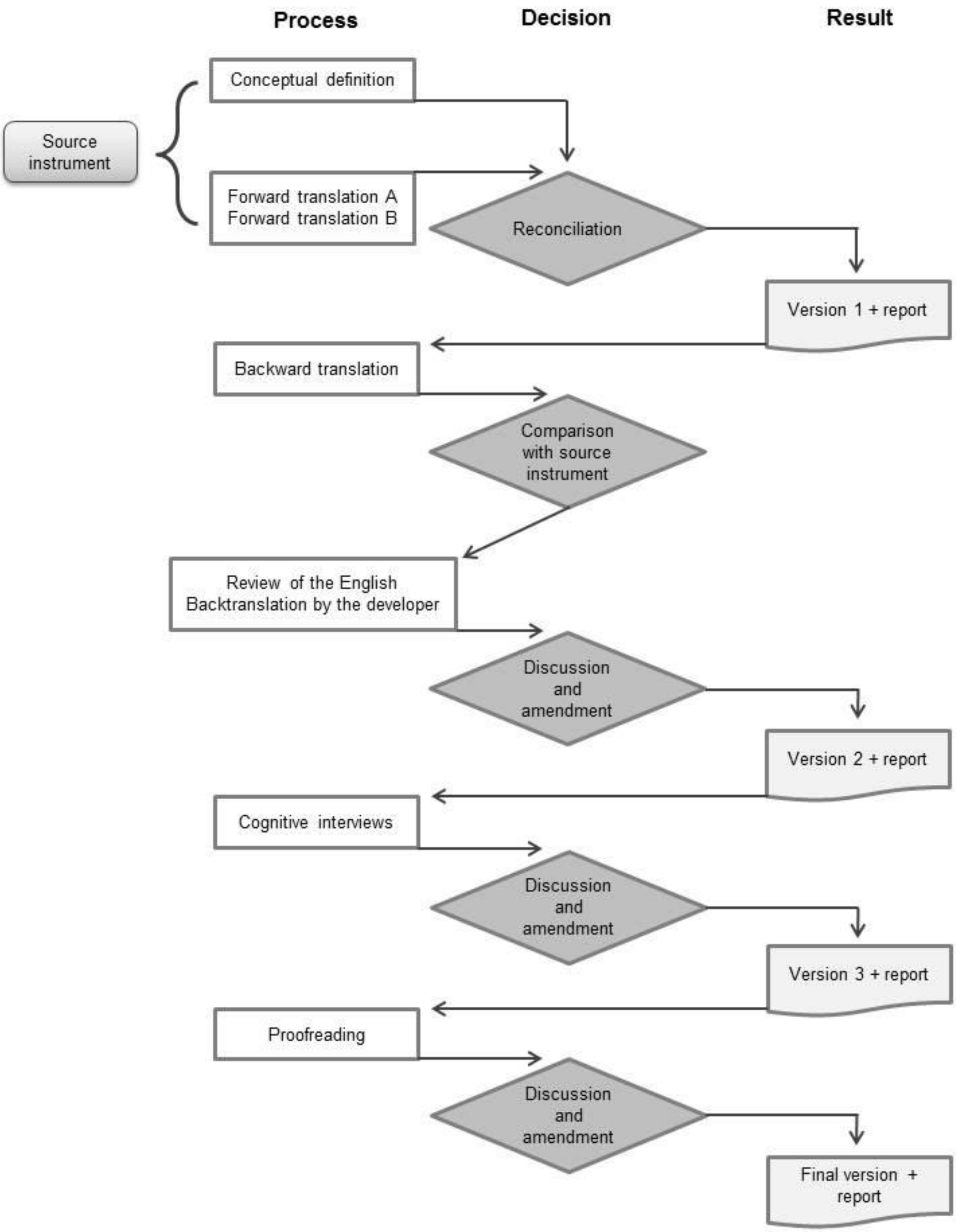


©Mapi Research Trust 2016. All rights reserved.
